# Identification of Marek’s Disease Virus VP22 Tegument Protein Domains Essential for Virus Cell-to-Cell Spread, Nuclear Localization, Histone Association and Cell-Cycle Arrest

**DOI:** 10.3390/v11060537

**Published:** 2019-06-08

**Authors:** Laëtitia Trapp-Fragnet, Katia Courvoisier, Sylvie Rémy, G. Le Pape, Fabien Loustalot, Caroline Denesvre

**Affiliations:** 1 INRA Val de Loire, UMR1282, Infectiologie et Santé Publique, Equipe Biologie des Virus Aviaires, F-37380 Nouzilly, France; katia.guyader@inra.fr (K.C.); sylvie.remy-delaunay@inra.fr (S.R.); fabien.loustalot@sensorion-pharma.com (F.L.); 2 Anastats, 14 rue de la Bretonnerie, F-37000 Tours, France; lepape.gilles@anastats.fr

**Keywords:** Alphaherpesvirus, VP22, virus spread, cell cycle, subcellular localization, histones, Marek’s disease virus, functional domains

## Abstract

VP22 is a major tegument protein of alphaherpesviruses encoded by the *UL49* gene. Two properties of VP22 were discovered by studying Marek’s disease virus (MDV), the Mardivirus prototype; it has a major role in virus cell-to-cell spread and in cell cycle modulation. This 249 AA-long protein contains three regions including a conserved central domain. To decipher the functional VP22 domains and their relationships, we generated three series of recombinant MDV genomes harboring a modified *UL49* gene and assessed their effect on virus spread. Mutated VP22 were also tested for their ability to arrest the cell cycle, subcellular location and histones copurification after overexpression in cells. We demonstrated that the N-terminus of VP22 associated with its central domain is essential for virus spread and cell cycle modulation. Strikingly, we demonstrated that AAs 174-190 of MDV VP22 containing the end of a putative extended alpha-3 helix are essential for both functions and that AAs 159–162 located in the putative beta-strand of the central domain are mandatory for cell cycle modulation. Despite being non-essential, the 59 C-terminal AAs play a role in virus spread efficiency. Interestingly, a positive correlation was observed between cell cycle modulation and VP22 histones association, but none with MDV spread.

## 1. Introduction

VP22 protein is a major tegument protein of *Alphaherpesvirinae* in the *Herpesviridae* family encoded by the *UL49* gene. VP22 protein is highly expressed in infected cells during the lytic cycle and is abundant in viral particles [1,2]. The deletion of *UL49* impacts viral replication differently according to virus species; it results in a total inhibition for gallid herpesvirus type 2, commonly named Marek’s disease virus (MDV) and human herpesvirus type 3 (or Varicella-Zoster virus, VZV) [3,4,5], a partial decrease for human herpesvirus type 1 (or herpes simplex type 1, HSV-1) and bovine herpesvirus type 1 (BoHV-1) depending on the cell type [6,7,8], and no effect for suid herpesvirus type 1 (or pseudorabies virus, PRV) [9]. For HSV-1 and VZV, replication impairment due to *UL49* deletion or a defect in VP22 post-translational modification has been attributed to a deficiency in morphogenesis and/or cell-to-cell spread [7,10]. For MDV, tagging VP22 with enhanced green fluorescent protein (EGFP) in N-terminal or C-terminal reduces viral spread in a different manner [11,12]. In addition to its role in assembly and spread, VP22 exhibits other functions. VP22 from HSV-1 and BoHV-1 is capable of intercellular trafficking [13,14]. HSV-1 VP22 was also shown to promote protein synthesis at a late stage of the lytic cycle [7,15] and to inhibit the interferon type I anti-viral response [16,17]. For MDV, we demonstrated that the cell cycle is arrested in the S phase upon infection and that MDV VP22 overexpression is sufficient to drive this phenotype in 90% of the transfected cells [18]. The cell cycle modulation activity of VP22 was also found to be conserved in its human orthologs encoded by HSV-1 and VZV [18].

VP22 protein interacts with numerous viral and cellular partners. For HSV-1 VP22, which is the most studied VP22, a dozen viral proteins from the tegument and the envelope have been reported as direct (for e.g., VP16, gE, gM, pUL16) or indirect partners [19,20,21,22,23]. Mutations in VP22 were shown to alter the localization of several viral proteins (ICP0, gE, gD, VP16, and VHS) [24]. The absence of VP22 abrogated or reduced the incorporation of viral proteins into HSV-1 particles, such as ICP0, ICP4 and gD [25]. Hew and collaborators suggested that HSV-1 VP22 may be a scaffold protein in the HSV-1 virion assembly, notably tegumentation and secondary envelopment [26]. Recent results suggested a similar role for VZV VP22/ORF9p [10,27]. VP22 not only binds to viral proteins but also to an astonishing number of cellular components: nucleic acids (DNA and RNA), histones, histone chaperone protein (TAF-1), chromatin, cellular membranes, and cytoskeleton proteins (microtubules and non-muscular myosin II) (reviewed in [28]). Recently, using a Y2H system, 31 cellular partners were identified as interacting with VZV VP22/ORF9p, including two histones and numerous proteins involved in organelle organization and intracellular transport [27]. In particular, the µ subunit of the adaptor protein complex 1 was shown to play an important role in VZV infectivity.

VP22 is a 241 to 304 AA-long protein and MDV VP22 is one of the shortest (249 AA). Experimental data indicated that HSV-1 VP22 homodimerizes [19,26,29]. Different isoforms of VP22 showed post-translational modifications, including phosphorylation by cellular and viral kinases, as well as ADP-ribosylation [10,30,31,32]. AA sequence alignments between several VP22 proteins revealed a conserved AA-central region of about 80 AA-long, also known as the core domain [26,29,33,34]. One long and two short alpha-helixes surrounding a ß-sheet were subsequently predicted in the VP22 core domain [35], and recently confirmed using X-ray cristallography at 1.9 Angström in HSV-1 VP22 [26]. Structural homologies between VP22 and histones were also suggested by several authors [35,36,37]. VP22 not only shares structural homologies between each other, but also functional ones. Indeed, by constructing MDV mutant viruses in which the *UL49* gene was replaced by another mardivirus (GaHV-3 or MeHV) or by the HSV-1 *UL49* gene, we previously showed that these three VP22 can rescue MDV spread [38].

Herein, our aim was to define domains of MDV VP22 essential for viral cell-to-cell spread and cell cycle arrest and to determine whether these functions are interconnected. For that, orthologous VP22 from varicelloviruses (VZV and PRV) and iltoviruses (gallid herpesvirus type 1 or infectious laryngotracheitis virus, ILTV) and different mutated VP22 were assessed for the two functions: (i) virus spread in the context of infection with recombinant MDV viruses; and (ii) ability to modulate the cell cycle after VP22 overexpression in avian cells. The link between the two functions was evaluated through statistical analyses of all data collected.

## 2. Materials and Methods

### 2.1. Cells and Original Plasmids

The chicken hepatocellular carcinoma cell line LMH was cultured on gelatin-coated flasks in William’s Medium E (Lonza) supplemented with 2 mM glutamine and 10% fetal bovine serum (FBS) at 37 °C in a 5% CO2 atmosphere. Chicken embryonic skin cells (CESCs) were prepared from 12-old day specific pathogen-free LD1 embryo and cultivated as previously described [33].

The pEGFP-UL49 MDV plasmid encoding MDV VP22 tagged with EGFP at its N-terminal extremity was previously described [11]. The genes encoding the PRV *UL49*, ILTV *UL49* and VZV *UL49/ORF9* were amplified from the PTD12 [9], pcDNA-ILTV49 [39] and pEGFP-9p plasmids, respectively (kindly provided by Pr L. Enquist, Dr W. Fuchs, Pr C. Sadzot). It is worth noting, for the sake of clarity in this manuscript, the terms VZV *UL49/ORF9* and VZV VP22/ORF9p will be used instead of ORF9 and ORF9p alone.

### 2.2. Multiple VP22 Proteins Alignment

Complete VP22 AA sequences from eleven alphaherpesviruses were aligned by using the DIALIGN multiple-alignment program on genomatix web site (http://www.genomatix.de/cgi-bin/dialign/dialign.pl). The Uniprot entries of each VP22 protein used are: MDV VP22 (Md5 strain) #Q9E6M7; GaHV3 VP22#A0A1P7U1I8; MeHV1 VP22 #Q9DHC2; HSV1 VP22 (strain 17) #P10233; HSV2 VP22 (strain HG52) #P89468; VZV VP22/ORF9p (strain Dumas) # P09272; PRV VP22 (strain Kaplan) #Q8QXN7; BoHV1 VP22 (strain Cooper) #P30022; EHV1 VP22 (strain Ab4p) #P28960; ILTV VP22 #A0A0K0K5Y8; PsHV1 VP22 #Q6UDL8.

### 2.3. MDV Bacterial Artificial Chromosome (BAC), Recombinant and Rescue Viruses

#### 2.3.1. Wild-Type MDV

This virus was obtained from the rRB-1B BAC, which corresponds to the repaired RB-1B 1272 BAC clone (kindly gifted by N. Osterrieder, Institut für Virologie/Freie Universität Berlin) [40]. This rRB-1B BAC is maintained in the GS1783, an Escherichia coli strain harboring a chromosomic I-SceI inducible enzyme (kindly provided by G. Smith, Northwestern University/Feinberg School of Medicine). All BACs and viruses described below were generated from this parental BAC. The progeny of this BAC was named herein rMDV.

#### 2.3.2. MDV Recombinant Viruses Encoding Orthologous and Chimeric *UL49*

All BAC mutants were generated by homologous recombination with the two-step Red-mediated recombination in *E. coli*, also named “en passant” mutagenesis [41]. Each shuttle plasmid was constructed as follows: (i) the MDV *UL49* gene was substituted by an orthologous gene (*UL49/ORF9* from VZV Dumas strain, *UL49* from PRV Kaplan strain or ILTV) or a chimeric MDV/ILTV *UL49* gene (MMI, MIM or IMM) (defined below in Section 2.4); and (ii) a specific I-SceI/kanamycin selection cassette was added, either in StuI or NheI site introduced in 5′ or 3′ of *UL49* gene. The first recombination step was performed as previously described [42]. For that, GS1783 bacteria were transformed by using a long restriction fragment overlapping the mutated *UL49* region and the Kanamycin selection cassette, obtained from each modified p48-50 StuNhe shuttle plasmid. Next, the selection cassette was totally excised by induction of I-SceI expression as well as the recombination machinery during a second recombination step. The six mutated BACs were verified by sequencing *UL47*-*UL50* region, in which the recombination occurred, between HpaI and XmnI restriction sites.

#### 2.3.3. MDV Recombinant Viruses Harboring MDV *UL49* Truncated at the 3′ End

The two BACs r22MDV^1–173^ and r22MDV^1–190^ were also generated by homologous recombination in *E. coli* using “en passant” mutagenesis [41]. These mutants were obtained by introducing two stop codons after codon 173 and codon 190 of the MDV *UL49* gene, respectively. Here, the first recombination step was performed after transformation of GS1783 bacteria by using a PCR fragment obtained with two sets of primers on pEPkan-S plasmid [41]: MUT∆173-1/MUT∆173-2 and MUT∆190-1/MUT∆190-2 for BAC r22MDV^1–173^ and BAC r22MDV^1–190^, respectively (Table 1). For these mutants, NheI and StuI sites are not present in 5′ and 3′ of *UL49*. The two BACs were verified by sequencing the region of recombination.

#### 2.3.4. Viral Progeny

Each BAC mutant (6 µg) was transfected into CESCs by nucleofection with a Nucleofector device (Lonza) and the Basic Mammalian Fibroblast kit (#VPI 1002, Lonza France, Levallois-Perret, France). Six days post-transfection, cell monolayers were observed for infection plaques. When plaques were obtained, the virus was passaged two times and virus stocks were frozen for subsequent analysis. For replicative and rescue mutants (see below), viruses used in this study did not exceed five passages. In the absence of plaques, cells were trypsinized and amplified once on fresh CESCs to allow slow viruses to grow. When viral progeny was not obtained after a second passage, a second transfection attempt was performed. If both transfection trials were negative, the mutant was considered non-replicative and assayed for a rescue virus.

#### 2.3.5. Rescue Viruses

For each non-replicative mutant, 6 µg of the BAC was co-transfected with 1 µg of the shuttle p48-50 StuNhe MDV *UL49* plasmid in CESCs by nucleofection. This plasmid is a pGEM-Teasy plasmid in which the *UL48*–*UL50* region of MDV RB-1B was cloned (4118bp), with two restriction sites inserted in 5′ and 3′ of *UL49*, StuI and NheI respectively. Rescue mutants were amplified as replicative viruses (see above).

### 2.4. VP22 Expression Vectors

MDV/ILTV VP22 chimera genes were generated on the basis of three regions in the VP22 proteins defined as: (i) the N-terminus region (AA1-96 of MDV VP22 and AA1-127 of ILTV VP22); (ii) the central domain conserved in VP22 encoded by herpesviruses (AA97-173 of MDV VP22 and AA128-204 of ILTV VP22); and (iii) the C-terminus region (AA174-249 of MDV VP22 and AA205-266 of ILTV VP22). Chimeras of the VP22 encoded gene (*UL49*) were obtained by “overlap-extend PCR” with primers, depicted in Table 1. Amplifications of MDV and ILTV *UL49* regions were performed from the pEGFP-UL49MDV and pcDNA-ILTV49 plasmids. Chimera constructs were named according to the ILTV and MDV regions appointed as “I” and “M”, respectively. Chimeric genes were thus called MMI, MIM and IMM, where, for example, the IMM gene encompassed the 5′-region of the ILTV *UL49* gene, the central part and the 3′-end of MDV *UL49*. Chimera genes obtained by PCR were inserted into the PCR2.1 TOPO TA cloning vector (ThermoFischer Scientific/Invitrogen, Waltham, Ma, USA). Eukaryotic expression vectors were obtained by cloning the *UL49* chimera genes in frame with EGFP at the BglII or XhoI sites in the pEGFP-C1 vector (BD Biosciences, Clontech, Mountain View, Calif, USA).

C-terminus truncations of VP22 encoded by MDV were obtained after PCR amplification from the pEGFP-UL49MDV [11], with primers listed in Table 1. The PCR products were inserted into PCR2.1 TOPO TA cloning vector and then sub-cloned into pEGFP-C1 vector in frame with EGFP at the BglII site.

Mutation of the MDV VP22 ß-strand region AA159-162 (sequence IKIT to AAAA) was introduced in the MDV *UL49* gene by “overlap-extend PCR”, with primers depicted in Table 1. Two PCR fragments were initially generated from the pEGFP-UL49 MDV plasmid, using the IKIT_fw/UL49_37R and IKIT_rev/UL49_37F’ primer pairs, including an overlapping internal sequence encompassing the mutation of interest. A second PCR step was then performed with 50 ng of each purified PCR product using the UL49_37F’/UL49_37R to obtain the UL49^AAAA^ gene that was cloned in frame with EGFP at the BglII site in the pEGFP-C1 vector.

All intermediate and final constructs were verified using DNA sequencing (Eurofins, MWG Operon, Les Ulis, France).

### 2.5. Detection of VP22 by Fluorescence Microscopy

CESCs transfected with the different MDV BACs or infected with replicative viruses were fixed with 4% paraformaldehyde (PFA) for 20 min at room temperature (RT), permeabilized with 0.5% Triton X-100 for 5 min at RT and blocked with PBS, 0.1% Triton X-100, 2% Bovine Serum Albumin (BSA). VP22 proteins were stained with the appropriate antibody: a rabbit polyclonal serum anti-PRV VP22 (PAS236) [43] (kindly provided by Pr L. Enquist, Department of Molecular Biology/Princeton University), a rabbit polyclonal serum anti-ILTV VP22 [39] (kindly provided by Dr W. Fuchs, Institute of Diagnostic Virology/Friedrich-Loeffler-Institut), a rabbit polyclonal serum anti-VZV VP22/ORF9p (kindly provided by Pr C. Sadzot-Delvaux, Virology and Immunology unit/GIGA Infection/University of Liege), mouse monoclonal antibodies B17 and/or D18 anti-MDV VP22 [33]. Next, an appropriate secondary antibody was used: a goat anti-rabbit or anti-mouse IgG (H + L) coupled to an Alexa Fluor dye 488 or 594 (Invitrogen). Subsequently, MDV infected cells were immunostained with a mouse monoclonal anti-VP5 MDV (F19) directly conjugated to a fluorochrome (Texas Red or Alexa Fluor^TM^ 488) (Invitrogen). Cell nuclei were counterstained with Hoechst 33342 dye (1:2000) (Invitrogen).

LMH cells transfected with EGFP-tagged MDV/ITLV VP22 chimeras and MDV VP22 truncated/mutated constructs were grown on glass coverslips and fixed at 24 or 48h post-transfection. Immunostaining was performed with monoclonal antibodies directed against tubulin to delineate the cytoplasm (#T9026, Sigma-Aldrich, St Louis, Mo, USA) at a dilution of 1:500 and detected with a goat anti-mouse IgG Alexa Fluor 594 (#A11005, Invitrogen Molecular Probes). Cell nuclei were counterstained with Hoechst 33342 dye. Fluorescence emitted from the EGFP-tagged VP22 protein was directly visualized and their cellular distribution was determined from a minimum of 100 transfected cells. Results are shown as percentage reflecting the nuclear and/or cytoplasmic distribution of the protein.

Microscopic observations were performed using an Axiovert 200M inverted epifluorescence microscope equipped with the Apotome imaging system (Zeiss, Göttingen, Germany). Images were captured with an Axiocam MRm camera and analyzed by using the Axiovision software (Zeiss).

### 2.6. Virus Cell-to-Cell Spread by Plaque Size Assay

CESCs (1.5 × 10^6^) grown in 6-well plates were infected with 200 pfu of parental, mutant or rescue viruses. At 4 or 5 days post-infection, cell monolayers were stained either with a cocktail of MDV mouse monoclonal antibodies (anti-VP22, -gB, -ICP4) or with a chicken hyper-immune serum from a MDV-infected bird followed with a goat anti-mouse Alexa Fluor 488 (#A11001, Invitrogen Molecular Probes) or a goat anti-chicken Alexa Fluor 488 (#A11039, Invitrogen Molecular Probes), respectively. The fluorescent plaques were photographed with the CCD camera depicted above, measured and analyzed as previously described [38]. At least 50 plaques were measured for each virus. The statistical analyses were performed as described below.

### 2.7. Detection of VP22 by Immunoblot

Whole cell lysates were prepared from monolayers of infected CESCs, non-infected CESCs or VP22 expressing LMH by resuspending the cells in laemmli 2× sample buffer. Solubilized proteins were separated on a 10% SDS-PAGE. Resolved proteins were transferred to nitrocellulose membrane, blocked in Tris 10 mM pH 8.25, NaCl 150 mM, Tween 0.2% containing milk 3% and subsequently incubated with the mouse monoclonal anti-MDV VP5 (F19), the rabbit polyclonal serum anti-PRV VP22, the mouse monoclonal anti-MDV VP22 (L13a), the rabbit polyclonal anti-ILTV VP22, a rabbit polyclonal anti-GFP (# 632593, Takara, Shiga, Japan) or a mouse monoclonal anti-GAPDH (MAB374, Millipore, Bedford, MA, USA) primary antibody. Subsequently, goat anti-mouse IgG (#A3562, Sigma) and anti-rabbit IgG alkaline phosphatase-conjugated antibodies (#A3687, Sigma) or goat anti-mouse IgG (#A4416, Sigma) and anti-rabbit IgG (#A0545, Sigma) peroxidase-conjugated antibodies were used. Alkaline phosphatase and peroxidase signals were revealed using NBT-BCIP (Zymed, South San Franscisco, Calif, USA) or Immobilon Western Chemiluminescent HRP substrate (P90720, Millipore), respectively.

### 2.8. Viral DNA Analyses of Rescue Mutants

Viral DNA was prepared from approximately 0.5-1 × 10^7^ infected CESCs with the different rescue mutants, as previously described [44]. Viral DNA was next amplified by PCR using Car6 and Car4 primers, purified and directly sequenced using MWG Operon with UL49_31F primer and UL49_5R primer to check for the presence of StuI site (Table 1). For the rescue viruses, the vDNA was amplified with recUL49_XmnI/ Car6 and the PCR fragment was sequenced with the Car4 primer.

### 2.9. Cell Cycle Analysis

The different eukaryotic expression vectors (pEGFP) expressing the MDV/ILTV VP22 chimeras or the truncated/mutated MDV VP22 proteins were transfected in triplicate into LMH cells by using Lipofectamine 2000, according to the manufacturer’s instructions (Invitrogen). At 24 or 48 h post-transfection, cells were fixed with 70% ethanol and the cell cycle was analyzed in transfected cells (EGFP-positive) by flow cytometry as previously described [18]. Of note, for VP22 mutants that did not associate to cellular structures, a fixation step with PAF 4% was needed in order to retain the proteins within cells and thus enabling the cell cycle analysis specifically in GFP-positive cells.

### 2.10. Histones Precipitation

LMH cells transfected with the mutant VP22 constructs were harvested 24 or 48h post-transfection and salt extraction of histones from chromatin was performed as previously described [18,45]. The proteins included in the extraction fraction were separated in a 10% SDS-PAGE gel and revealed with colloidal Coomassie blue staining. Images of the gels were captured with the Fusion-FX7 imaging system (Vilber Lourmat, Marne-la-Vallée, France) and the level of VP22 proteins found in the histone extract was estimated using the Bio-profil 1D++ software (ChemiSmart 5000) as a ratio of VP22 signal relative to total protein input (for each lane).

### 2.11. Statistics

All graphs and statistics, except for Figure 6 (Spearman correlation test and principal component analysis), were performed using the GraphPad Prism software version 5.02 (San Diego, USA). Spread data are presented with Tukey boxes. A Kruskal-Wallis test was used to compare differences in multiple groups and the Mann-Whitney (two-tailed) test was used to compare variables between two groups (for e.g., mutant vs rMDV). Cell cycle and localization data are presented as means and standard error of the mean (±SEM). The Kruskal-Wallis test was used to compare differences in multiple groups and the Mann-Whitney (two-tailed) test was used to compare non-parametric variables between two groups. *p* values <0.05 were considered statistically significant as indicated in the figure legends.

A Spearman correlation test was performed using R software [46] to evaluate whether a correlation exists between MDV spread and cell cycle arrest. This analysis was done only with VP22 mutants or orthologues, for which data concerning these 2 parameters were available. For each VP22, the functionality was graded from 1 to 3: 1, when abolished; 2, when below 70% of the MDV VP22 activity; and 3, when above 70% of the MDV VP22 activity. Principal component analysis (PCA) was performed to evaluate the relationship between cell cycle, nuclear localization, and histone association using the R software. VP22 mutants without missing data were used. For each VP22, the functionality was graded as above.

## 3. Results

### 3.1. PRV UL49 Partially Cis-Complements MDV Spread Whereas VZV and ILTV UL49 Do Not

**Figure 1:**
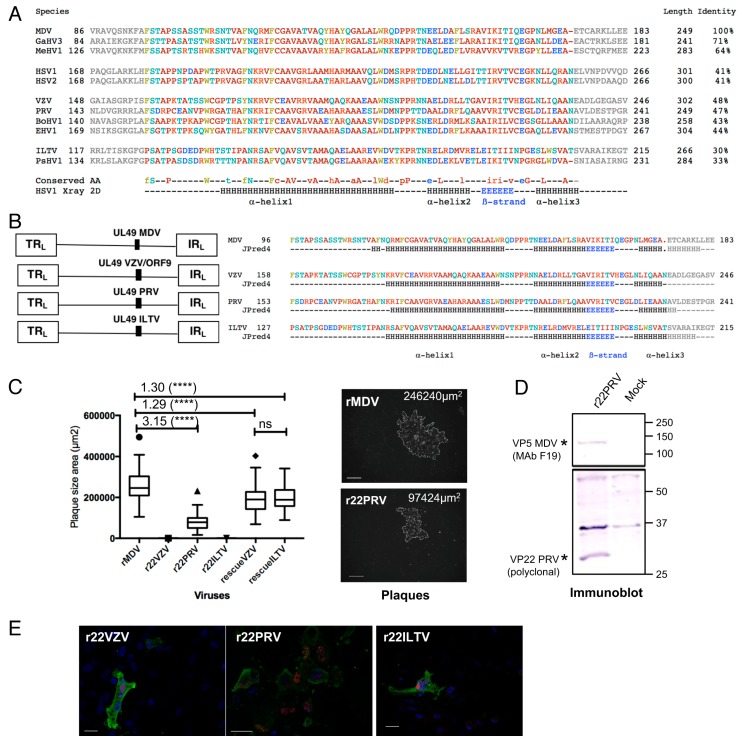
Construction and characterization of recombinant Marek’s disease virus (MDV) mutants harboring an orthologous *UL49* gene derived from Varicello or Iltoviruses. (**A**) Multiple alignments of the VP22 central region from 11 alphaherpesvirus species. From this alignment, the conserved region of 79–80 AAs (colored letters) was selected with 10 AAs at the N- and C-terminal edges (grey letters). The letter color codes are: basic AAs (red), non-polar AAs (brown), uncharged polar AAs (turquoise), acidic AAs (blue), aromatic AAs (green). For each position, a consensus sequence is given below (conserved AAs) with capital letters when the AA is conserved for all VP22s, and lowercase letters when the AA is conserved in 6 or more VP22 sequences. The HSV-1 2D structure determined by-X ray by Hew and collaborators is given for comparison, with the position and length of the 3 alpha-helixes and of the ß-strand (adapted from [26]). Alpha-helixes are identified as “H” and ß-strands as “E”. The AA length of each VP22 as well as the % of identity in the conserved core region compared to the MDV sequence is given on the right, in the “identity” column. (**B**) On the left panel, a schematic representation of the parental RB-1B strain (rMDV) and of the three derived recombinant MDV genome (r22VZV, r22PRV, r22ILTV) is shown. On the right panel, the core AA sequences of VP22 MDV, Varicella-Zoster virus (VZV), pseudorabies virus (PRV) and infectious laryngotracheitis virus (ILTV) are provided, with the predicted secondary structure obtained with JPred4 (http://www.compbio.dundee.ac.uk/jpred4) below. (**C**) Spread of mutated and rescue viruses in culture by plaque assay. On the left panel, the box-and-whisker plot is representative of two independent experiments. r22PRV replicated and spread 3.15-fold less than rMDV, whereas r22VZV and r22ILTV did not. The rescue VZV and ILTV replicated, with a mild attenuation compared to rMDV (statistical differences are indicated: **** *p* < 0.0001; ns: not significant). Pictures (right panel) show a plaque of r22PRV and rMDV in fluorescence microscopy after immunostaining of MDV antigens 5 days post-infection. Bars, 200 µm. (**D**) Characterization of the replicative r22PRV. The expression of PRV VP22 was detected in a lysate of r22PRV-infected CESCs by immunoblotting using PAS236 antiserum. In parallel, MDV VP5 expression was verified with the F19 monoclonal antibody. The black stars indicate PRV VP22 (about 30kDa) and MDV VP5. (**E**) Expression of the orthologous VP22 into CESCs transfected with BAC r22VZV, r22PRV, or r22ILTV by fluorescence microscopy. A VP22 signal was detected for each recombinant mutant, including the two non-replicative mutants (r22VZV and r22ILTV) with specific anti-VP22 antibodies, indicating that the orthologous VP22 were expressed. Bars, 20 µm.

A phylogenetic tree constructed with 10 VP22s from the four alphaherpesvirus genuses indicated that MDV VP22 is more closely related to PRV or VZV than to HSV-1 and ILTV VP22 (Figure S1). Multiple alignments with these VP22 proteins showed that the VP22 core domain is 78 to 79 AA-long and has a different position in each VP22 (Figure 1A, Figure S1). The core region of VP22 encoded by VZV (AA158–236), PRV (AA153–231), and ILTV (AA127–205) show 48%, 47%, and 30% AA identity, respectively, with the MDV VP22 core region (AA96–173) (Figure 1A). Using the JPred4 prediction tool, all core regions of VP22 show conserved secondary structures (3 alpha-helices and a ß-strand) [47] (Figure 1B), which is in accordance with HSV-1 VP22 X-ray structure [26]. Hence, to assess whether VP22 from varicello- and iltoviruses share functional homologies with MDV VP22, we constructed three MDV recombinant viruses from the very virulent rRB-1B genome in which we substituted MDV *UL49* with *UL49/orf9* from VZV, PRV, or ILTV (Figure 1B). The three final BACs r22VZV, r22PRV, and r22ILTV were transfected independently into primary CESCs by nucleofection. A viral progeny was obtained only with r22PRV containing the PRV *UL49* gene (Figure 1C). A plaque size assay showed that r22PRV spread was reduced 3.15-fold compared to the parental rRB-1B (named herein rMDV) (Figure 1C). PRV VP22 expression in infected cells was validated by immunoblot and fluorescence microscopy (Figure 1D,E). We also showed using RT-qPCR that the level of expression of two immediate early genes (*ICP4* and *ICP27*) was similar upon r22PRV and rMDV infection, whereas expression of UL48 and of three late genes (*US3*, *UL13* and *US8*/gE) was down-regulated (Figure S2A). This result indicates a global reduction in viral replication and spread. In order to exclude that non-expected mutations outside *UL49* led to r22PRV attenuation, we generated a rescue mutant (rescuePRV) which harbored a spread similar to the parental rMDV (Figure S2B). No replicative virus was obtained after transfection of r22VZV and r22ILTV BACs, despite several attempts (Figure 1C). However, VP22 and the major capsid VP5 from MDV were detected in a few isolated transfected cells by fluorescence microscopy, showing that the viral cycle was initiated while formation of infectious virus failed (Figure 1E). In order to verify that the orthologous VZV and ILTV *UL49* genes were indeed responsible for the null-phenotype, rescue mutants were assessed by cotransfection of r22VZV or r22ILTV BACs with the shuttle plasmid p48-50 StuNhe encoding the full *UL48*–*UL50* locus (see Material and Methods). Both rescue mutants were replicative, with a mild but significant reduction in spread (Figure 1C), which was associated with the enzymatic sites introduced at the 5′ and 3′ ends of *UL49* in the course of the mutagenesis. Together, these results demonstrated that *UL49* of VZV and ILTV do not cis-complement MDV for cell-to-cell spread, contrary to PRV *UL49* which partially restores it. The fact that PRV and VZV VP22 share 48% and 47% identity, respectively, in the central region with MDV VP22 but are not functionally equivalent for MDV spread indicates that the homology in the central region of the protein is not sufficient to explain the cis-complementation of MDV VP22 function. Therefore, some non-conserved AAs in the core region or in the VP22 N- and/or C-terminus regions may also play a role in this function.

### 3.2. MDV Spread Is Supported by MDV VP22 N-Terminus Associated to Its Core Domain

**Figure 2:**
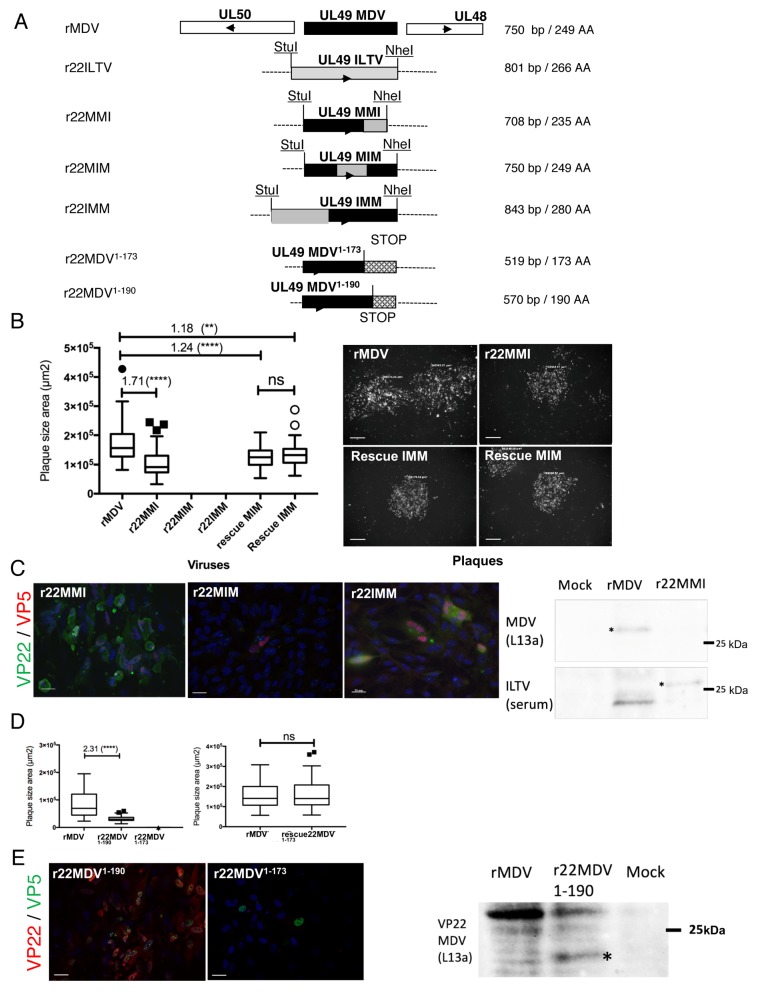
Construction and characterization of recombinant MDV mutants harboring a MDV/ILTV chimeric *UL49* gene or a truncated MDV *UL49* gene. (**A**) Schematic representation of the mutated MDV genomes: r22MMI, r22MIM, r22IMM, r22MDV^1–190^, and r22MDV^1–173^. For each chimeric *UL49* gene (MMI, MIM, and IMM), one region of the MDV *UL49* gene (5′ end, central, or 3′ end) was replaced by the homologous region of the ILTV *UL49* gene. For the truncated MDV *UL49* gene, two stop codons were inserted after codon 190 or 173 of MDV *UL49*, in order to partially or totally shorten its C-terminus. (**B**) Spread of mutated viruses in culture measured by plaque size assay. The graph on the left is representative of two independent experiments. r22MMI replicated and spread 1.7-fold less than rMDV, whereas rMIM and rIMM did not. The rescue rMIM and rIMM replicated, with a mild attenuation compared to rMDV (statistical differences are indicated: **** *p* < 0.0001; ** *p* = 0.0037; ns: not significant). Pictures on the right show infection plaques observed with rMDV, r22MMI, and the rescue viruses (IMM and MIM) by fluorescence microscopy after staining plaques with antibodies to MDV or ILTV antigens. Bars, 200 µm. (**C**) Expression of the chimeric MDV/ILTV VP22 into CESCs transfected with BAC r22MMI, r22MIM, or r22IMM. By fluorescence microscopy, expression of MMI VP22 was detected with mouse monoclonal antibodies recognizing MDV VP22 (green). Expression of the MIM and IMM VP22 was detected using ILTV anti-serum (green). For the three mutants, MDV VP5 major capsid antigen was stained with F19 mouse monoclonal antibody (red). Bars, 20 µm. By Western blot (right panel), MMI VP22 was detected only with ILTV anti-serum and not with anti-MDV VP22 antibody (L13a). (**D**) Spread analysis of the r22MDV^1–190^, r22MDV^1–173^ and rescue 22MDV^1–173^ mutated viruses in culture by plaque size assay. r22MDV^1–190^ spread 2.3-fold less than rMDV, whereas r22MDV^1–173^ was not replicative. In contrast, the rescue 22MDV^1–173^ mutant was fully replicative, like rMDV (statistical differences are indicated: ns: not significant). (**E**) Expression of the truncated MDV VP22. With fluorescence microscopy, monoclonal antibodies (B17 and/or D18) were used to detect the truncated VP22 (red). These antibodies detected VP22^1–190^, but not VP22^1–173^, although a VP5 signal (green) was detected for both MDV mutants, including the non-replicative r22MDV^1–173^. Bars, 20 µm. Using Western blot analysis (right panel), VP22^1–190^ was detected with L13a antibody (at 20 kDa, shown by an asterix), but faintly compared to the full-length VP22 (27 kDa).

To explore the role of the N- and C-terminus regions of MDV VP22 in viral spread, we constructed three MDV recombinant genomes in which the N-terminus, conserved-central core, or C-terminus regions of MDV *UL49* were substituted with the corresponding region of ILTV *UL49*. ILTV *UL49* was chosen because of its avian origin and its failure to cis-complement MDV in spread. The AA sequences of the N- and C-terminus domains of both VP22 were determined according to the position and AA sequence of the core domain, as described above. This led to r22MMI, r22MIM, and r22IMM (Figure 2A) mutated genomes harboring distinct MDV/ILTV chimeric *UL49* genes. The three BACs were transfected independently into CESCs. A viral progeny was obtained with r22MMI, but not with r22IMM and r22MIM. It is worth noting that a few small plaques containing less than ten infected cells were observed 5 days post-transfection with r22MIM and r22IMM by using MDV VP5 antibody, but these small plaques never yielded a sustainable infection after passage onto fresh cells. r22MMI showed a 1.7-fold reduction in spread efficiency compared to the parental rMDV (Figure 2B). Its VP22 was detected with monoclonal antibodies specific to MDV by fluorescence microscopy and with the anti-ILTV serum by Western blot analysis (Figure 2C). Upon infection with r22MMI, mRNA viral genes (*ICP4, ICP27, UL48, UL13, US3, US8/gE*) were expressed at a similar level to rMDV (Figure S2A). To exclude that mutations outside *UL49* contribute to the attenuated phenotype, we generated a rescue MMI virus and confirmed that it spread like the parental rMDV (Figure S2B). Cells infected with r22IMM or r22MIM expressed MDV VP5, but the expression of chimeric VP22 proteins was detected with confidence only in r22IMM-infected cells with the ILTV VP22 anti-serum (Figure 2C) and not with B17 and D18 MDV VP22 antibodies. The expression of both *UL49* (IMM and MIM) was verified by reverse transcriptase PCR 72 h post-transfection (Figure S3). Rescue mutants of r22IMM and r22MIM were generated and their spread was assessed by plaque assay (Figure 2B). Both rescue mutants spread like rMDV, indicating that no mutation outside *UL49* gene is impairing virus replication. Therefore, IMM and MIM VP22 do not cis-complement MDV for replication and spread, unlike MMI VP22. Thus, in term of viral spread, our data showed that the N- and core domains of MDV VP22 are not substitutable with the orthologous domains of ILTV in contrast to the C-terminus domain, which could share homologous function or be dispensable.

### 3.3. The Putative Alpha-3 Helix of MDV VP22 Core Is Extended in the C-Terminus Region and Is Essential for MDV Spread

To explore whether the C-terminus region of MDV VP22 is dispensable or not for MDV spread, we constructed the r22MDV^1–173^ recombinant virus, encoding a VP22 deleted of the whole C-terminus domain (from the edge of the core domain to the end of the protein) (Figure 2A). No viral progeny was obtained with this mutant (Figure 2D). After transfection, however rare VP5-positive cells were detected using fluorescence microscopy, but none of them were VP22-positive using two different monoclonal antibodies directed to MDV VP22 (D18 and B17) (Figure 2E). As the expression of UL49^1–173^ RNA was confirmed by reverse transcriptase PCR (Figure S3), the non-detection of VP22^1–173^ by immunofluorescence reveals either the disappearance of the conformational epitopes or a rapid degradation of the shortened VP22. The rescue mutant r22MDV^1–173^ was generated and was shown to spread as the parental rMDV (Figure 2D, on the right panel). These results clearly indicated that the C-terminus of MDV is essential for MDV spread. As the C-terminus of ILTV which partially complements this defect (as shown above) is very poorly homologous in the AA sequence, we suspected the possible role of a conserved structure between the C-term of MDV and ILTV. Looking back at the VP22 sequence alignments and at the 2D predictions, we observed that the alpha-3 helix of the core domain of both VP22 (MDV and ILTV) is predicted to be longer than for the other VP22 (incl. HSV-1). For MDV VP22, the alpha-helix extends in the C-terminus domain and ends at Leu180. We therefore generated a second truncated mutant, the r22MDV^1–190^, in which the VP22 truncation started ten AA downstream of the end of the putative long alpha-3 helix. A viral progeny was obtained with r22MDV^1–190^ (Figure 2D). This mutant virus replicated 2.3 times less than the parental rMDV in CESCs and expressed VP22 as shown by fluorescence microscopy and Western blot analysis (Figure 2E). Upon infection with r22MDV^1–190^, mRNA viral genes (ICP4, ICP27, UL13, gE) were expressed at similar level as rMDV or mildly reduced (for e.g., UL48), reflecting the virus cell-to-cell spread attenuation and possibly a weak replication defect. All together, these results indicate that the AAs 174–190 are essential for MDV spread and support the need for the integrity of an extended alpha-3 helix, ending in the core domain. In contrast, AAs 191–249 are dispensable for MDV spread, but favor it.

### 3.4. The N-Terminus and Core Domains of MDV VP22 Are Essential for VP22 Cell Cycle Modulation Activity, Nuclear Localization, and Histone Association

**Figure 3:**
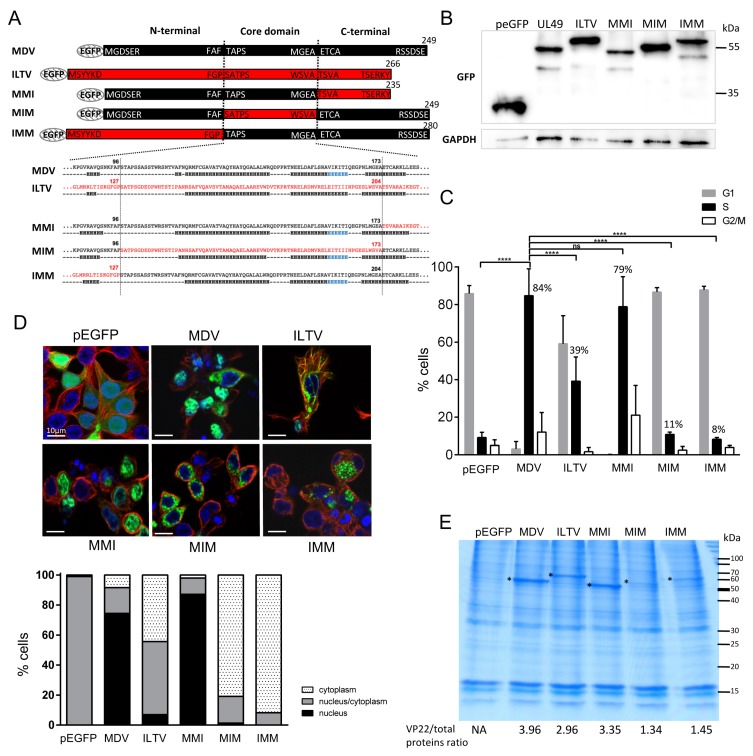
Cell cycle modulation, subcellular localization and histones association of MDV/ILTV VP22 chimeras. LMH cells were transfected for 48 h with pEGFP-C1 and pEGFP eukaryotic expression vectors encoding the ITLV VP22 and MDV/ILTV VP22 chimeras. (**A**) MDV/ILTV VP22 chimera are schematized with the AA sequence junctions indicated. Boxes in black and red correspond to VP22 AA sequences of MDV origin and of ILTV origin, respectively. Below the AA sequences of the core domain and the junction are the corresponding secondary structures predicted using JPred4. (**B**) The expression of the MDV ITLV and chimera proteins was verified in LMH whole-cell extract by immunoblot using an anti-GFP antibody. GAPDH expression was determined as an internal loading control. (**C**) Cell cycle analysis. The percentage of cells in G0/G1, S and G2/M phases of the cell cycle is represented as bars (means ± SEM) (statistical differences compared to *UL49* S-phase results are indicated: **** *p* < 0.0001; ns: not significant). At 48 h post-transfection, transfected cells were harvested. DNA content in pEGFP-positive cells was analyzed by flow cytometry after staining with propidium iodide. Eighty-four percent of the cells transfected with MDV VP22 were arrested in S-phase compared to 39% with ILTV VP22. Among the MDV/ILTV VP22 chimeras, only MMI showed this property, whereas MIM and IMM lost it. (**D**) Subcellular localization of VP22. Transfected cells were fixed with PFA 4% and observed by fluorescence microscopy after staining the cytoplasm using an anti-α-tubulin antibody (red). Nuclei were counterstained with Hoechst 33342 (blue) and EGFP (green) was visualized directly. A representative example of the results obtained is shown. Bars, 10 µm (upper panel). The nuclear/cytoplasm distribution of EGFP was estimated on an average of 100 cells and results are represented as stacked bars (lower panel). (**E**) Histones were extracted in high salt conditions from transfected cells. Extracts were separated using SDS-PAGE. Proteins were directly stained in the gel with colloidal coomassie blue. (*) indicates the presence of the parental and chimeric VP22 proteins. The values indicated under each gel correspond to the percentage of recombinant VP22 protein detected relative to the total protein input loaded in each lane.

We previously showed that MDV infection leads to an intra-S cell cycle arrest and that overexpression of MDV VP22 in proliferative avian cells drives this phenotype. This property of VP22 is conserved in the VP22 encoded by human alphaherpesviruses HSV-1 and VZV [18]. In order to determine the domains of VP22 involved in this function and its potential link with MDV spread, we next assessed the ability of the VP22 mutants depicted above to modulate the cell cycle. First, ILTV VP22 fused with EGFP at its N-terminus extremity (Figure 3A) was overexpressed in LMH cells. The expression of the protein was verified by immunoblot (Figure 3B) and the DNA content of LMH cells was analyzed in GFP + cells by flow cytometry at 48 h post-transfection (Figure 3C). This showed that ILTV VP22 promotes cell cycle arrest in S-phase, but to a lower extent than MDV VP22, since only 39% of cells expressing ILTV VP22 accumulated in S-phase as opposed to 84% of cells overexpressing MDV VP22. Since we previously observed that the regulation of the cell cycle by MDV VP22 depends on its nuclear localization and its ability to associate with histones [18], we also explored whether ILTV VP22 could be altered in these characteristics. The subcellular distribution of ILTV VP22, as assessed by fluorescence microscopy (Figure 3D), was nuclear in 7% of the cells and nucleo-cytoplasmic in about 50%. In comparison, MDV VP22 was nuclear in 74% of the cells, and found in both nucleus and cytoplasm in 17% of the cells. By using a high-salt histones extraction protocol, we showed that ITLV VP22 was included in the histones fraction at a slightly lower level than MDV VP22, and thus shared this property with MDV VP22 (Figure 3E). Therefore, despite being capable of association with histones, VP22 ILTV had a poor exclusive nuclear localization and a mild cell cycle arrest activity.

We took advantage of these differing properties between MDV VP22 and ILTV VP22 to delineate the domains of VP22 involved in the intra-S arrest by testing chimeric MDV/ILTV VP22 proteins described above. The three *UL49* chimeric genes (MMI, MIM, and IMM) were fused with the EGFP gene at their 5′ end in the pEGFP-C1 eukaryotic expression vector (Figure 3A). These constructs were transfected in LMH cells (Figure 3B) and the cell cycle, the cellular localization, and the histone association of these chimeras were analyzed at 48 h post-transfection. Strikingly, we observed that the MMI VP22 construct was the only chimera capable of inducing an intra-S phase arrest at a rate (79% of cells blocked in S-phase) that was comparable to the rate of wild-type MDV VP22 protein (84%) (Figure 3C). In contrast, MIM and IMM VP22 had completely lost their cell cycle modulation ability (11% and 8% respectively, like the pEGFP empty vector). Moreover, MMI VP22 showed an exclusive nuclear localization in 87% of the cells, in contrast to MIM and IMM VP22 which were mainly located in the cytoplasm with punctate distribution (in 80 to 90% of the cells) (Figure 3D). In addition, MMI VP22 was the only chimera clearly detectable in the histones fraction at a similar level to MDV VP22, the IMM and MIM VP22 chimeras being 2- to 3-fold less retained in this fraction (Figure 3E), despite their higher expression in cells detected by Western blot (Figure 3B).

All together, these results substantiate a link between the S-phase promoting activity of VP22, its nuclear localization and its ability to copurify with histones. Moreover, it appears that the combination of the N-terminus and core domains of the MDV VP22 protein is mandatory for these properties of VP22. Replacement of the N-terminus or core domain by ILTV sequences abolished the ability of MDV VP22 to arrest cells in S-phase and to be targeted to the nucleus. These findings suggest that the C-terminus region of MDV VP22 is either non-essential for these VP22 functions or is cis-complemented by the orthologous region of ILTV, like previously shown for MDV spread.

### 3.5. The Putative Extended Alpha-3 Helix of MDV VP22 Is Also Essential for VP22 Cell Cycle Modulation Activity

**Figure 4:**
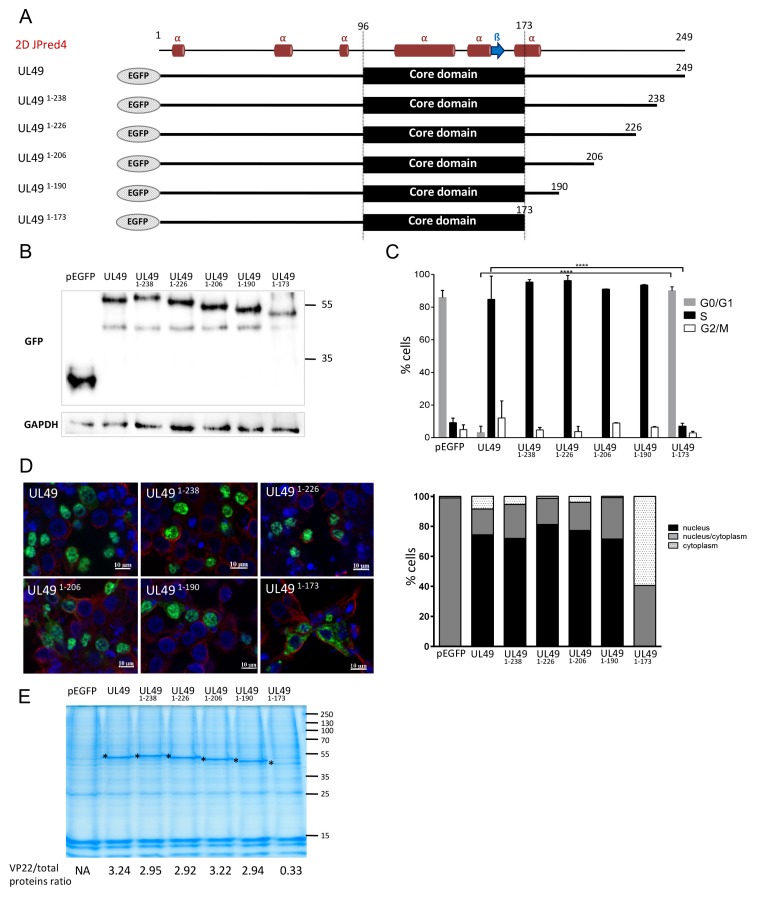
Cell cycle modulation and subcellular localization of MDV VP22 proteins truncated at the C-terminus. LMH cells were transfected for 48 h with pEGFP-C1 and pEGFP eukaryotic expression vectors encoding MDV VP22 protein truncated at its C-terminus. (**A**) MDV VP22 truncated proteins are schematized, as well as the secondary structure of the full MDV VP22 predicted with JPred4. (**B**) Expression of the VP22 truncated proteins was detected in LMH whole cell extract using immunoblot using an anti-GFP antibody. GAPDH expression was determined as an internal loading control. (**C**) Cell cycle analysis performed by flow cytometry in transfected EGFP-positive cells. The percentage of cells in the G1, S, and G2/M phases of the cell cycle is reported as bars (statistical differences were only found between the native *UL49* and UL49^1–173^ as indicated (**** *p* < 0.0001); no-significant difference was found for the other truncated *UL49*. (**D**) Subcellular localization of the truncated VP22 was established at 48 h post-transfection by direct visualization of EGFP protein (green) by fluorescence microscopy. The cytoplasm was demarcated using an anti-tubulin antibody (red) and the nuclei were observed with Hoechst 33342 counterstaining (blue). A representative image is shown (bars, 10 µm) (upper panel) and the nuclear/cytoplasm distribution of EGFP estimated an average of 100 cells is represented as stacked bars (lower panel). (**E**) Histones were extracted in high salt conditions from transfected cells. Extracts were separated using SDS-PAGE. Proteins were directly stained in the gel with colloidal coomassie blue. (*) indicates the presence of the native and truncated VP22 proteins. The values indicated under each gel correspond to the percentage of recombinant VP22 protein detected relative to the total protein input loaded in each lane.

To clarify the role of the C-terminus domain of VP22 with respect to its cell cycle modulation and nuclear localization properties, we generated a set of VP22 mutants serially truncated at the C-terminus extremity: two VP22 described above (UL49^1–173^ andUL49^1–190^) and three new ones (UL49^1–206^, UL49^1–226^ and UL49^1–238^) (Figure 4A). These last three were performed to define the minimal sequence which is fully functional for cell cycle arrest and also to evaluate the presence of putative nuclear localization signal (NLS). Indeed, the C-terminus of BoHV-1 VP22 was shown required for nuclear localization [36]. All mutants were cloned in fusion with EGFP at their N-terminus and overexpressed in LMH cells. At 48 h post-transfection, the subcellular localization of VP22 was determined by fluorescence microscopy and the presence of VP22 in histones extracts was studied. In addition, the cell cycle was analyzed on GFP + cells. Expression of VP22 proteins harboring deletions from AAs 174 to 249 was verified using immunoblotting (Figure 4B). All truncated proteins were able to induce an intra-S cell cycle arrest as efficiently as the wild-type MDV VP22 protein (in more than 80% of the cells), with the exception of the VP22 truncated of the whole C-terminus domain (UL49^1–173^), which was totally defective for the cell cycle modulation function (Figure 4C). In addition, this VP22 mutant was distributed either exclusively in the cytoplasm (59% of the cells) or in both the nucleus and the cytoplasm (41%). The UL49^1–173^ protein had a fluffy appearance, reminiscent of aggregate proteins. In comparison, the full MDV VP22 protein and the other truncated VP22 mutants were mainly detected in the nucleus (in more than 70% of the cells). Finally, UL49^1–173^ was poorly detected in the histone fraction whereas the others truncated VP22 were found at the same level as the full length VP22 (Figure 4E). These results indicate that the region (AAs 174–190) is essential to the intra S-phase arrest activity of VP22, its nuclear targeting, and histones association and sufficient to restore a fully functional protein for these three properties. In contrast, the downstream C-terminus region (AAs 191–249) is totally dispensable for them.

### 3.6. The 4-AA Predicted in the ß-Strand of the MDV VP22 Core Domain Are Essential for VP22 Cell Cycle Modulation Activity and Nuclear Localization

**Figure 5:**
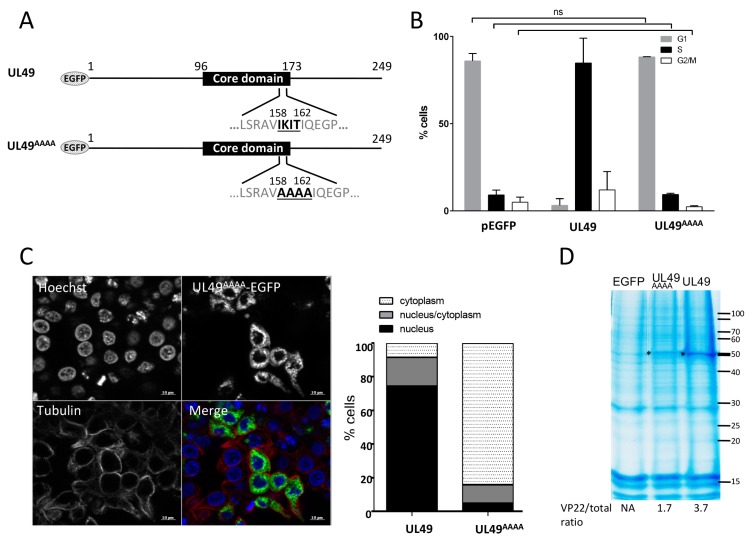
Role of the AAs 159-162 of the core domain of VP22 on cell cycle modulation and subcellular localization. LMH cells were transfected for 48 h with pEGFP-C1 and pEGFP eukaryotic expression vectors encoding the native MDV VP22 protein and the UL49^AAAA^ recombinant protein harboring a 4AA mutation (159-162 IKIT→AAAA) within the core domain. (**A**) UL49^AAAA^ mutant protein is schematized. (**B**) Cell cycle analysis performed by flow cytometry in transfected EGFP-positive cells. The percentage of cells in the G1, S, and G2/M phases of the cell cycle is reported as bars. As indicated, no significant difference (ns) in cell cycle modulation was found upon pEGFP-C1 and pEGFP-UL49^AAAA^ expression (all results obtained from the native *UL49* expression were statistical different (*p* < 0.0001) from those obtained with UL49^AAAA^ or the empty vector pEGFP-C1). (**C**) Cellular localization of the UL49^AAAA^ mutant was established at 48 h post-transfection by direct visualization of EGFP (green) by fluorescence microscopy. The cytoplasm was demarcated by using an anti-tubulin antibody (red) and the nuclei were observed with Hoechst 33342 counterstaining (blue). A representative image is shown (bars, 10 µm) (left panel) and the nuclear/cytoplasm distribution of EGFP estimated on an average of 100 cells is represented as stacked bars (right panel). (**D**) Histones were extracted in high salt conditions from transfected cells. Extracts were separated by SDS-PAGE. Proteins were directly stained in the gel with colloidal coomassie blue. (*) indicates the presence of the native MDV VP22 and UL49^AAAA^ proteins. The values indicated under each gel correspond to the percentage of recombinant VP22 protein detected relative to the total protein input loaded in each lane.

As shown in Figure 1, a ß-strand of six AA (V158 I159 K160 I161 T162 I163) is predicted in MDV VP22, like in other VP22. Hew et al. [26] proposed that the conserved AAs in the ß-strand located in the core domain of HSV-1 VP22 might be important for protein function [26]. To test this assumption for MDV VP22, we mutated 4-AA of this region into alanine (159-162 IKIT→AAAA) (Figure 5A). The *UL49* mutated recombinant gene UL49^AAAA^ was fused with EGFP in the pEGFP-C1 vector and overexpressed in LMH cells. At 48 h post-transfection, the UL49^AAAA^ had lost its ability to arrest the cell cycle in S-phase (only 9,3%) compared to the native MDV VP22 protein (Figure 5B). Although the mutant protein showed an exclusive cytoplasmic localization in 84% of the transfected cells (Figure 5C), we nevertheless could detect the protein in the histone fraction, but at half the extent of the native VP22 (Figure 5D). These results demonstrated that this 4AA-region predicted to be located in the ß-strand of the core domain is mandatory for MDV VP22 cell cycle modulating activity and its nuclear localization.

### 3.7. Relationship between VP22 Functions

**Figure 6:**
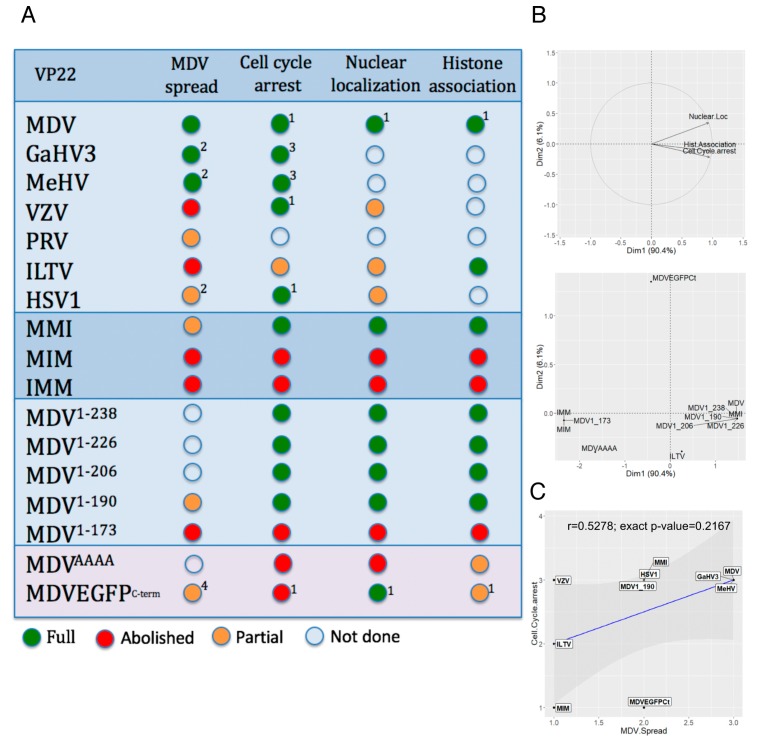
Correlation between VP22 functions. (**A**) Summary of the functionality results obtained with VP22 orthologues or mutants in MDV context (spread) or after VP22 overexpression in LMH cells (cell cycle arrest, histone association and nuclear localization). The functions were quoted as follows: abolished (red dot), partial below 70% activity (orange dot), above 70% activity to full (green dot). Results from previous reports were included: ^1^ HSV-1 VP22, VZV VP22 and MDVEGFP^C-term^ [18], ^2^ r22HSV1 [38], ^3^ MeHV VP22, ^4^ MDVEGFP^C-term^ that encodes a VP22 fused to EGFP protein at its C-terminus [12]. (**B**) Principal component analysis (PCA) between nuclear localization, histone association and cell cycle arrest for twelve VP22 mutants. The upper graph shows a correlation between the three functions. The lower graph indicates the VP22 mutants that contribute mostly to the first factor and to the second factor. (**C**) Spearman correlation test between MDV spread and cell cycle arrest for ten VP22. Each VP22 is positioned on the graph. Grade 1, abolished function; grade 2, partial function (<70% activity), grade 3, ≥70% activity.

The functionalities of the VP22 mutants analyzed in this study and in three past studies are summarized in Figure 6A. First, to examine the relationship between nuclear localization, histone association, and cell cycle modulation, we performed a PCA. For that, we used all VP22s for which we had complete data (n = 12): MDV, ILTV, MMI, MIM, IMM, MDV^1–238^, MDV^1–226^, MDV^1–206^, MDV^1–190^, MDV^1–173^, MDV^AAAA^, and MDVEGFP^c-term^. The direction of the three variables projected on the plane are close and in the same directions (Figure 6B, upper graph) indicating that nuclear localization, histone association, and cell cycle modulation are positively correlated. The first factor shows the contrast between four of the VP22s (IMM, MIM, MDV^1–173^ and MDV^AAAA^) with low levels for the three variables and a group of eight others, including MDV VP22, with high levels (Figure 6B, lower graph). Only one of the VP22s, MDVEGFP^C-term^, contributed to the second factor, because of a particular profile, with low cycle arrest and high nuclear localization. Second, a correlation test was performed using the Spearman method to explore the relationship between cell cycle arrest and MDV spread functions of VP22. This analysis was performed only with VP22 proteins, which were suspected not to be aggregated after overexpression and for which we had data for both functions (*n* = 10): MDV, GaHV3, MeHV, VZV, ILTV, HSV1, MMI, MIM, MDV^1–190^, and MDVEGFP^c-term^. This test indicated that cell cycle arrest and MDV spread functions of VP22 are not correlated (r = 0.5278; exact *p*-value = 0.2167) (Figure 6C).

## 4. Discussion

The present study provides new insights into the domains carrying the different functionalities of the MDV VP22 protein. Herein we showed by using orthologous VP22 that PRV *UL49* cis-complements MDV spread at 32%, whereas VZV *UL49* and ILTV do not. Previously, we reported a partial complementation of MDV spread with *UL49* from other mardiviruses (above 70% for GaHV-3 and MeHV) and with HSV-1 *UL49* (14%) [38]. Due to the homologies in AA sequence and 2D structure restricted to the VP22 core region, these results suggest that the core region is the main support of the VP22 function in MDV spread. However, efficient *UL49* cis-complementation was not systematically linked to the highest % of AA in the core region, suggesting a role of non-conserved AAs in this function. This can be explained by the fact that VP22 does not act alone in viral spread but probably through its interaction with other viral partners. For HSV-1, several viral proteins were identified to interact with VP22 and were essential for replication and spread, such as VP16 and gE [19,20,29,48,49]. Moreover, interestingly, a single or double mutation in the HSV-1 VP22 core region was sufficient to abolish VP22 interaction to both viral proteins [21,49]. Interestingly, for MDV, mutants lacking VP16 or gE are attenuated or non-replicative, respectively (Pasdeloup et al., manuscript in preparation) [3,50]. Although for MDV, no viral partners of VP22 has been reported yet, an alteration in the binding of VP16 and/or gE to VP22 appears as an interesting hypothesis to explain the spread defect that we observed with rMDV harboring orthologous VP22. The characterization of these protein interactions, including the binding domains, remains to be determined.

Our approach using an MDV/ITLV VP22 chimera pointed out that the MDV core region has to be associated to its homologous N-terminus region to be functional in MDV cell-to-cell spread, nuclear localization, histones association, and cell cycle arrest. In contrast, by serial truncations of the C-terminus, we could demonstrate that the 59 C-terminal AAs of MDV VP22 (AAs 191-249) are dispensable for all functions assessed in culture. Despite not being essential for MDV spread, AAs 191-249 contribute to the spread efficiency as demonstrated by the spread attenuation of r22MDV^1–190^.

Moreover, we showed that a VP22 with the whole C-terminus (r22MDV^1–173^ or UL49^1–173^) deleted is completely defective for virus spread and cell cycle arrest, demonstrating that AAs 174–190 of MDV VP22 are essential for these functions. The exact role of these 17 AA-region (AAs 174–190) remains to be determined. We suspect that a deletion of this region might severely impact the structure of the MDV VP22 protein and notably the integrity of the alpha-3 helix. This helix was predicted at the C-terminus extremity of the core domain for most VP22 and demonstrated by crystallography in HSV-1 VP22 between AAs 250-257 (NLLQRANE) [26]. Our secondary 2D structure predictions indicated that this helix might be longer for MDV VP22 (AAs 168–180) and for ILTV VP22 (AAs 199–212) compared to HSV-1 VP22 (Figure 1B) [26]. We suspect that the truncation from AA 173 disrupts the MDV alpha-3 helix, possibly causing VP22 misfolding and subsequently a loss of function. The fact that VP22 functions (both spread and cell cycle arrest) are restored by adding the ILTV C-terminus in fusion to MDV UL49^1–173^ (r22MMI and MMI) is in accordance with this interpretation. We also propose that the integrity of the extended alpha-3 helix conserved between MDV and ILTV VP22 is essential for proper protein folding and functionality (MDV spread, nuclear localization, histone association, and cell-cycle arrest).

Moreover, the MMI VP22 is more efficient in restoring MDV spread than the truncated UL49^1–190^ protein. Even if the C-terminus of MDV VP22 is poorly homologous in AA sequence compared to that of ILTV (as shown on Figure S1), we cannot eliminate the possibility that another molecular determinant in the C-terminus of ILTV contributes to the spread, in addition to the extended alpha-3 helix.

Interestingly, we demonstrated that the four AAs 159-162 (IKIT) of MDV VP22 play a key role in cell cycle arrest, as well as in nuclear localization and histones association. This 4-AA sequence is conserved in the core domain of all VP22 (I-K/R-I-T/L/I) and is part of a 8-AA ß-strand identified in HSV-1 VP22 by X-rays [26]. This ß-strand is able to form a ß-sheet when the two anti-parallel ß-strands interface, and contributes to HSV-1 VP22 dimerization [19,26,29]. Although MDV VP22 dimerization has not yet been proven, we cannot not exclude the possibility that AAs 159-162 (IKIT) of MDV VP22 might also be involved in this process, and be indirectly essential for VP22 functions. Even if the mutations did not abolish the ß-strand according to JPred4 software, we make the hypothesis that the removal of the arginine and the threonine affect the stability of the interface between the two anti-parallel ß-strands.

In the present study, we demonstrated that the nuclear localization is necessary for cell cycle arrest mediated by VP22, but not sufficient. Indeed, all of our VP22 mutants (either chimeric, or truncated) that are able to arrest the cell cycle like MDV VP22 showed a predominant nuclear localization in more than 70% of the transfected cells. In contrast, VP22 mutants that had lost this function (MIM, IMM, UL49^1–173^, and UL49^AAAA^) were totally or predominantly cytoplasmic (≥ 60%). In addition, ILTV VP22, which partially arrests the cell cycle (39%), showed partial nucleo/cytoplasmic localization, but very little exclusive nuclear localization. This result indicates that the nuclear localization of VP22 is essential but not sufficient for cell cycle arrest and that these VP22 mutants exhibit other functional defects, as discussed above. Such a phenotype was also previously observed for MDV VP22 fused to EGFP at its C-terminus, MDVEGFP^C-term^ [18]. Interestingly, in silico prediction did not reveal any classic or non-classic NLS within the AA sequence of MDV VP22. Only few reports have depicted an NLS in VP22 orthologues [35,36,51]. Sequence alignment of MDV VP22 with VP22 orthologues only shows a very low homology to the NLS sequences depicted previously. We thus speculate that nuclear targeting of MDV VP22 may be mediated by an association of VP22 with cellular proteins such as histones, as was previously evocated for BoHV-1 VP22 [36]. We demonstrated that MDV VP22 is highly retained within the histone fraction. In addition, our data and PCA analyses strongly support a relationship between the capacity of VP22 to mediate cell cycle arrest and its ability to substantially copurify with histones, confirming the hypothesis that we proposed earlier [18]. VP22 recombinant proteins that triggered cell cycle arrest (MMI, ILTV, UL49^1–238^, UL49^1–226^, UL49^1–206^, and UL49^1–190^) were copurified with histones in LMH cells. The presence of VP22 mutants in the histones fraction is in accordance with the ability of several VP22 (incl. MDV) to bind histones, chromatin, and/or mitotic chromosomes [36,37,52,53,54,55,56] [27]. Conversely, VP22 mutants that lack the capacity to modulate the cell cycle (IMM, MIM, UL49^1–173^, and UL49^AAAA^) were not found in the histones fraction or slightly associated.

Lastly, statistical analyses indicate that the cell cycle arrest ability of VP22 is not correlated to the spread function, suggesting that these two functions are independent. Such a result was surprising, because cell cycle arrest was demonstrated to favor replication of several herpesvirus [57]. Further exploration of this topic is warranted.

## 5. Conclusions

Our results indicate that the N-terminus region associated with the core region (AAs 1–190) is essential for MDV cell-to-cell spread, nuclear localization, histones association, and cell cycle arrest. In contrast, the C-terminus region (AAs 191–249) is dispensable for all functions, but favors MDV cell-to-cell spread. In addition, we showed that AAs 174–190 are essential for MDV VP22 functionality, possibly due to the presence of a longer alpha-3-helix (compared to HSV-1), whose interruption could lead to protein misfolding. Interestingly, we demonstrated that the IKIT motif (AAs 159–162) highly conserved in the core domain of all VP22 and located in the predicted ß-strand plays a key role in nuclear localization, histones association, and cell cycle arrest. Our data also showed that the VP22 cell cycle arrest ability correlates with its substantial histones association, but not with the viral spread function.

## Figures and Tables

**Table 1:** Primers used in this study.

Name	Use	Sequence (5′ to 3′)
Hpa5FL49ILTV	chimera construct	AGTTAACATGTCTTACTACAAAGATCTC
IMI_1R	chimera construct	TGAAGGAGCCGTACTAGGACCAAATCCCTTAGAGA
IMI_1S	chimera construct	AATATTATATCTTAGTTATCAGGCCTATGTCTTACTACAAA
IMI_2S	chimera construct	AGTACGGCTCCTTCATCAGC
IMI_2R	chimera construct	TCTTGCCACCGAAGTGGCTTCCCCC
IMI_3S	chimera construct	ACTTCGGTGGCAAGAGCAAT
IMI_3R	chimera construct	GGGCTGTTCCTATGCTAGCCTAGTACTTT
IMM_2R	chimera construct	TGTTCCTATGCTAGCTTATTCGCTATCACTG
MIM_1R	chimera construct	TGAGGGTGTAGCACTGAAAGCGAATTTATTACTTTG
MIM_2S	chimera construct	AGTGCTACACCCTCAGGGGATGAAGACCCATGGC
MIM_2R	chimera construct	TTTGCGGGCACAGGTTTCGGCAACAGACCATAAAGA
MIM_3S	chimera construct	GAAACCTGTGCCCGCAAACTATTGGAAGAGTCTGGAT
MMI_1S	chimera construct	TACTGTTTAATATTATATCAGGCCTATGGGGG
NheI3RUL49ILTV	chimera construct	GGGCTAGCCTAGTACTTTCGTTCGGATGTC
MM1_173	*UL49*MDV truncation	AAGCTTAGGCTTCCCCCATCAAATTTGGAC
MM1_190	*UL49*MDV truncation	AAGCTTAGGGTTCCCCTGGGATAATCCAGA
MM1_206	*UL49*MDV truncation	AAGCTTAGGTGTACGTTCAGATTTGGTTGTACG
MM1_226	*UL49*MDV truncation	AAGCTTAGGGTTATGTGTACGATGAGATC
MM1_238	*UL49*MDV truncation	AAGCTTAGGTGAATGATGGCGACGCGAAGTTG
IKIT_fw	*UL49*MDV mutation	TTCTTTCCAGAGCTGTCgcagctgcagctATTCAAGAGGG
IKIT_rev	*UL49*MDV mutation	ATTTGGACCCTCTTGAATagctgcagctgcGACAGCTCTGG
UL49_37F’	*UL49*MDV truncation and sequencing	CCAGATCTTTGGGGGATTCTGAAAGGCGG
UL49_37R	*UL49*MDV mutation	CCAAGCTTTTATTCGCTATCACTGCTACG
UL49_31F	sequencing	CCCTCGAGATGGGGGATTCTGAAAGGCGG
UL49_5R	sequencing	CGTCATCATATGCAGAGGG
Car4	sequencing and PCR on rescue	GGATGTCTATAAAAGACGAC
Car6	sequencing and PCR on rescue	TGTTTAAAGAGGAGTGGTAA
recUL49_XmnI	PCR on rescue	TAGTAACAGACGGAGCAACC
MUT∆173-1	BAC r22MDV^1–173^	gataatccagactcttccaatagtttgcgggcacagCtAttAggcttcccccatcaaatttggAGGATGACGACGATAAGTAGGG
MUT∆173-2	BAC r22MDV^1–173^	aaattaccattcaagagggtccaaatttgatgggggaagccTaaTaGctgtgcccgcaaactaCAACCAATTAACCAATTCTGATTAG
MUT∆190-1	BAC r22MDV^1–190^	tcagatttggttgtacgttcagatttggactttacgttActAgttcccctgggataatccagAGGATGACGACGATAAGTAGGG
MUT∆190-2	BAC r22MDV^1–190^	ccgcaaactattggaagagtctggattatcccaggggaacTagTaacgtaaagtccaaatcCAACCAATTAACCAATTCTGATTAG

## Supplementary Materials

The following are available online at https://www.mdpi.com/1999-4915/11/6/537/s1, Figure S1: Multiple alignment and phylogenetic tree with ten full-length alphaherpesviruses VP22 AA sequences, Figure S2: Further characterization of r22PRV and r22MMI attenuation by quantifying viral mRNA expression and rescue mutants spread, Figure S3: VP22 mRNA expression in CESCs transfected with rMDV and mutant BACs.
