# Supplementary material for: Identification of Marek’s Disease Virus VP22 Tegument Protein Domains Essential for Virus Cell-to-Cell Spread, Nuclear Localization, Histone Association and Cell-Cycle Arrest
Source: Viruses. 2019 Jun 8;11(6):537. doi: 10.3390/v11060537 (PMC6631903; doi:10.3390/v11060537)
Supplement: Supplementary file 1 [file viruses-11-00537-s001.zip › Figure S1_revised2(viruses-456072).pdf]

A. Multiple alignment

```
VP22ILTV        -------------------------------MSYYK------------------------   5
VP22MeHV1       MGDSEGRKYERR--------RPS-----VYHSHQDGTGGTDGTTRRKSTRSLQSPPRDDY   47
VP22GaHV2       MGDSERRKSERR--------RSL-----GYPSAYDDVSIPARRPSTRTQR----------   37
VP22GaHV3       MGDSDRRKSSRR--------RST-----MRTSPDNSAHISSTRARRDSSK----------   37
VP22HSV1        MTSRRSVKSGPR-----------EVPRDEYEDLYYTPSSGM-------------------   30
VP22HSV2        MTSRRSVKSCPR-----------EAPRGTHEELYYGPVSP--------------------   29
VP22HHV3        MASSDGDRL--------------CRS----NAVRRKTTPSYSGQYRTARRSVVVGPPDDS   42
VP22PRV         MSSSRKTRVAADETASGARRRAGSASRT------RTTAPAAATPRRPSAY-------DDY   47
VP22BoHV1       ----------------------------------------MARFHRPSEDE------DDY   14
VP22EHV1        MSDTWR------------RRRSGCNDANATEELVYSTVRSDHRQRRPSRGTFV-MRENDL   47

VP22ILTV        ----------DLSEEAKKYHDDTRR-RRDTTTRRPKPPQFEHPWSG-------RRTSPYL   47
VP22MeHV1       LHAS------------------------RVTSNRHAR-----------------------   60
VP22GaHV2       ------------------------------------------------------------   37
VP22GaHV3       ------------------------------------------------------------   37
VP22HSV1        ------------ASPDSP-PDTSR---RGALQTRSRQ---RGEVRFV-----QYDESDY-   65
VP22HSV2        ------------ADPESP-RDDFR---RGAGPMRARP---RGEVRFL-----HYDEAGY-   64
VP22HHV3        DDSLGYITTV-GADSPSPVYADLYFEHKNTTPRVHQPNDSSGSEDDFEDIDEVVAAFRE-   100
VP22PRV         DDGF-------SYRSAPSYDDDGYYGYDGYGSSRAPR------------AA-KVTPAAA-   86
VP22BoHV1       EYSDLWVRENSLYDYESG-SDDHVYEELRAATSGPEPSGRRASVRACASAA-AVQP----   68
VP22EHV1        YDKQSVSKENDLYESASP-NDDKVYTRRGMSTAAHYR-DSEHIYETCEGDE-FYDACEY-   103

VP22ILTV        DLDRGSDSDSSEGDYGHPSR-------AHYSR-DHTAPPQG---------RRSPPMESFR   90
VP22MeHV1       ---------------SPPRAELPRSTRRQSAHHAESSPPEER--------PGPSDHRSLQ   97
VP22GaHV2       ---------------------------------NLNQDDLSK--------HGPFTDHPTQ   56
VP22GaHV3       ---------------------------------NESPDRIS------------PPSHSLQ   52
VP22HSV1        ALYGGSSSEDDEHPEVPRTRRPVSGAVLSGPGPARAPPPPAGSGGAGRTP-TTAPRAPRT   124
VP22HSV2        ALYRDSSSDDDESRDTARPRRSASVAGSHGPGPARAPPPPGGPVGAGGRSHAPPARTPKM   124
VP22HHV3        ARLR--------------H-ELVEDAV-------------------------------YE   114
VP22PRV         SR---------------------ASTGAKSASAAKT--PASAAKT-A---RS--------   111
VP22BoHV1       -AAR--------------GRDRAAAAGTTVAAPAAA--PARRSSSRA---SSRPPRAAAD   108
VP22EHV1        SLIG--------------GGKLSTSNGRQSPAKAQP--PPR---GAA---AAPPPRVPTR   141

VP22ILTV        KETTPKEEPP------QSKRG---------WNPDNHCAG--LMRRLTISKGFGPSATPSG   133
VP22MeHV1       RRKSVKEVEPAN----TSKSS--------SIPLGQRARP--GVRAVQK-NKFMFSSAPTS   142
VP22GaHV2       KHKSAKAVSEDVSST---TRG--------GFTNKPRAKP--GVRAVQS-NKFAFSTAPSS   102
VP22GaHV3       RRRSVKIERKDSSSE-TQRGE--------SLSSKVRAKP--GARAIEK-GKFAFSTTPAS   100
VP22HSV1        QRVATKAPAAPAAETTRGRKSAQPESAALPDAPASTAPTRSKTPAQGLARKLHFSTAPPN   184
VP22HSV2        TRGAPKASATPATDPARGRRPAQADSAVLLDAPAPTASGRTKTPAQGLAKKLHFSTAPPS   184
VP22HHV3        N---PLSVEKPSRSFTKNAAVKPKL-----EDSPKRAPP--GAGAIASGRPISFSTAPKT   164
VP22PRV         A---PAAAPAATTTTTTTAAAEPAA-----RRASTRAAP--GENLDVGRRRLAFSDRPCE   161
VP22BoHV1       P---PVLRPATRGS--SGGAGAVAV-----GPPRPRAPP--GANAVASGRPLAFSAAPKT   156
VP22EHV1        P---PTRAAA------TSTTPRQQD-----CAPKQRASP--GVNSIKSGKGLAFSGTPKT   185
                                                    .              :  *  *

VP22ILTV        DEDPWHTSTIPANRSAFVQAVSVTAMAQAELAAREVWDVTKPRTNRELRDMVRELEITII   193
VP22MeHV1       RTSHWKSNTVAFNQHVFCCAVAAVARYHAFRGALALWNKEPPRTDEQLEDFLVRAVVKVT   202
VP22GaHV2       ASSTWRSNTVAFNQRMFCGAVATVAQYHAYQGALALWRQDPPRTNEELDAFLSRAVIKIT   162
VP22GaHV3       ATSTWRSNTLVYNERIFCGAVAAVAQYHAYRGALSLWRRNAPRTNAELEEFLARAIIKIT   160
VP22HSV1        PDAPWTPRVAGFNKRVFCAAVGRLAAMHARMAAVQLWDMSRPRTDEDLNELLGITTIRVT   244
VP22HSV2        PTAPWTPRVAGFNKRVFCAAVGRLAATHARLAAVQLWDMSRPHTDEDLNELLDLTTIRVT   244
VP22HHV3        ATSSWCGPTPSYNKRVFCEAVRRVAAMQAQKAAEAAWNSNPPRNNAELDRLLTGAVIRIT   224
VP22PRV         ANVPWRGATHAFNKRIFCAAVGRVAEAHARAAAESLWDMNPPTTDAALDRFLQAAVVRIT   221
VP22BoHV1       PKAPWCGPTHAYNRTIFCEAVALVAAEYARQAAASVWDSDPPKSNERLDRMLKSAAIRIL   216
VP22EHV1        PKSQWYGATHLFNKNVFCAAVSRVAAAHASDAASALWDLNPPKTNEDLDRFLKAAAIRIL   245
                    *   .   *.  *  **   *   *  .*   *    * .:  *  ::    : :
```

```
VP22ILTV        INPGESLWSVATSVARAIKEGTPITHELLQKRPSKPP---TRRKTEDGTR--KSSSRPSQ    248
VP22MeHV1       VREGPYLLEEAESCTQRFMEETGLGSADNKPKSRS-------------------GRSERD    243
VP22GaHV2       IQEGPNLMGEAETCARKLLEESGLSQGNENVKSKS-------------------ERTTKS    203
VP22GaHV3       IQEGANLLDEAEACTRKLSEESGLSPDMGNPKSRS-------------------QYGKR-    200
VP22HSV1        VCEGKNLLQRANELV----NPDVVQDVDAATATRGRS--AASRPTERPRAPARSASRPRR    298
VP22HSV2        VCEGKNLLQRANELV----NPDAAQDVDATAAARGRP--AG-RAAATARAPARSASRPRR    297
VP22HHV3        VHEGLNLIQAANEAD----LGEGASVS-----KRGHNRKTGDLQGGMGNE-PMYAQ-VRK    273
VP22PRV         VCEGLDLIEAANAVL----DESTPGRK-----GKVYK-----------------------    249
VP22BoHV1       VCEGSGLLAAANDIL----AARAQRPA-----ARGST--------SGG------ESRLRG    253
VP22EHV1        VCEGAQLLEVANSTM----ESTPDGYA-----AAGPN--------GYDRR-PRTASRRRS    287
                :  *  *   *

VP22ILTV        P----KPEH-FP-----------PPRRKTSERKY--------------- 266
VP22MeHV1       VES---GEGSFNS---GARRPIAIARLVSSAQSFADSPGERTSDSE--- 283
VP22GaHV2       ERTRRGGEIEIKSPDPGSHRTHNPRTPATSRRHHSSARGYRSSDSE--- 249
VP22GaHV3       -----DGDESTPVDKRDRRSKTPGRAPTTSRRHYSSSRGNYSSESE--- 241
VP22HSV1        P-----VE----------------------------------------- 301
VP22HSV2        P-----LE----------------------------------------- 300
VP22HHV3        PKSR------TDTQTTGRITN--------------RSRARSASRTDTRK 302
VP22PRV         ------------------------------------------------- 249
VP22BoHV1       ERARP-------------------------------------------- 258
VP22EHV1        LKCKPPADDFFDDTNSG-------------------------------- 304
```

B. Phylogenetic tree

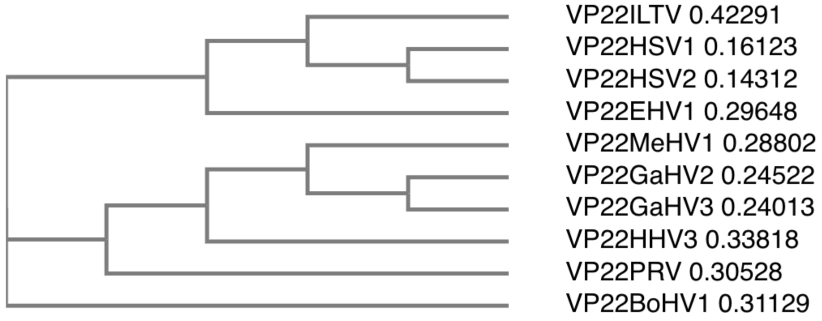

Figure S1: The multiple alignment of ten full-length alphaherpesviruses VP22 AA sequences was performed with CLUSTAL O(1.2.4) on www.ebi.ac.uk website. The region high-lighted in yellow corresponds to the core domain of MDV VP22 (named above as GaHV2 VP22). The phylogenetic tree constructed based on the multiple alignment is a neighbour-joining tree without distance corrections. This tree shows that MDV VP22 is closely related to PRV or VZV than to HSV-1 and ILTV VP22.
