# Supplementary material for: Identification of Marek’s Disease Virus VP22 Tegument Protein Domains Essential for Virus Cell-to-Cell Spread, Nuclear Localization, Histone Association and Cell-Cycle Arrest
Source: Viruses. 2019 Jun 8;11(6):537. doi: 10.3390/v11060537 (PMC6631903; doi:10.3390/v11060537)
Supplement: Supplementary file 1 [file viruses-11-00537-s001.zip › Figure S2_revised2(viruses-456072).pdf]

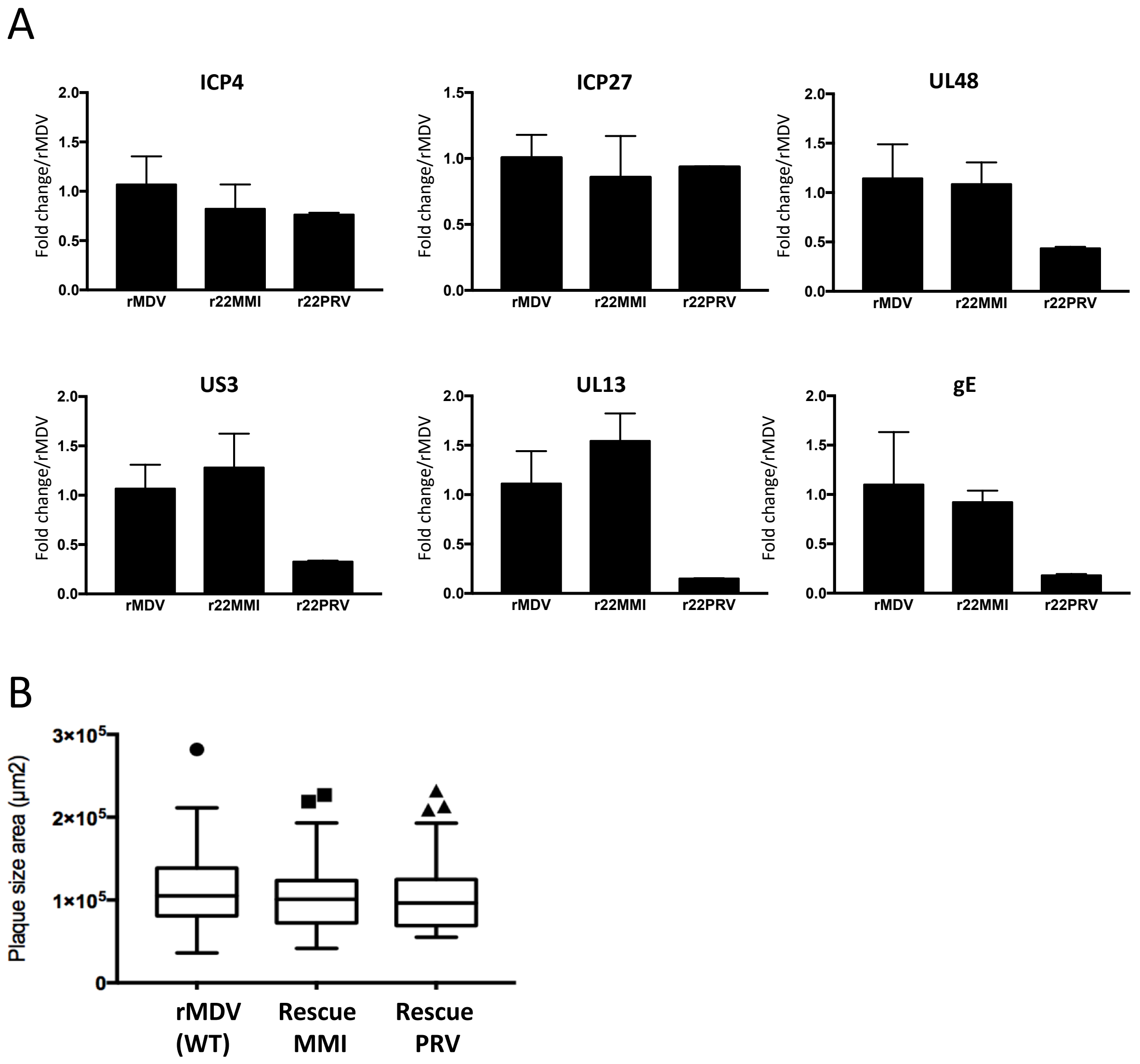

Figure S2. Further characterization of r22PRV and r22MMI attenuation by quantifying viral mRNA expression and rescue mutants spread. (A) To determine whether the expression of key viral proteins (*e.g.* VP16 and gE) could be compromised upon r22PRV and r22MMI infection and could thus contribute to viral attenuation, we infected CESCs with the parental rMDV virus, the r22MMI or r22PRV viruses. At 96hpi, total RNAs were extracted using the RNeasy minikit (Qiagen) and RT-qPCR were performed as previously described (Trapp-Fragnet *et al.*, 2014). Primer pairs used for detection of ICP4, ICP27 (*UL54*), UL48 (VP16), UL13, US3 were depicted previously (Trapp-Fragnet *et al.*, 2014). Expression of gE was determined with the primer pair: For5′CGTTTTGGGTCAAGCAAGGG3′/Rev5′ CCGCCGTCGAGACATTGATA3′. In CESCs infected with r22MMI, all viral genes tested were similarly expressed as upon rMDV infection. For r22PRV infection, a downregulation of the expression of UL48 and of late viral genes (US3, UL13 and gE) was detected, whereas the expression of immediate early genes (ICP4 and ICP27) was similar to rMDV. This overall decrease in UL48 and late viral genes expression indicates that VP22 PRV is globally attenuated in replication and spread.

(B) To establish whether the attenuated phenotype of r22PRV and r22MMI was not due to additional mutations outside of the *UL49* gene, we constructed rescue mutants. For both BACs (r22PRV and r22MMI), we replaced the entire *UL49* mutant genes (*UL49* PRV or *UL49* MMI) by the Kan-S cassette by homologous recombination in *E. coli*, leading to the BAC r22PRV:Kan and BAC r22MMI:Kan. For that, a unique recombination step was performed with a PCR fragment obtained from pEPkan-S plasmid using the two following primers (mutΔ49_IsceI_F:5′gaaggtgcacttgttcatatcttactgtttaatattatatcttagttatcAGGATGACGACGATAAGTAGGG3′;mutΔ49R_Kan_R:5′ggatgtctataaaagacgacttacttgcagtagtagggctgttcctatgcaaccaattaaccaattctgattag3′). Next, the procedure described for non-replicative rescue mutant was followed (see material and methods, section 2.3.5). Briefly, BAC r22PRV:Kan or the BAC r22MMI:Kan were cotransfected with the shuttle p48-50 StuNhe MDV *UL49* plasmid in CESCs. Viral spread was assayed and compared to parental rMDV virus (WT). Both rescue mutants spread like the WT (no statistical differences), indicating that no mutations outside of the *UL49* gene is reducing virus spread.
