# Supplementary material for: Identification of Marek’s Disease Virus VP22 Tegument Protein Domains Essential for Virus Cell-to-Cell Spread, Nuclear Localization, Histone Association and Cell-Cycle Arrest
Source: Viruses. 2019 Jun 8;11(6):537. doi: 10.3390/v11060537 (PMC6631903; doi:10.3390/v11060537)
Supplement: Supplementary file 1 [file viruses-11-00537-s001.zip › Figure S3_revised2(viruses-456072).pdf]

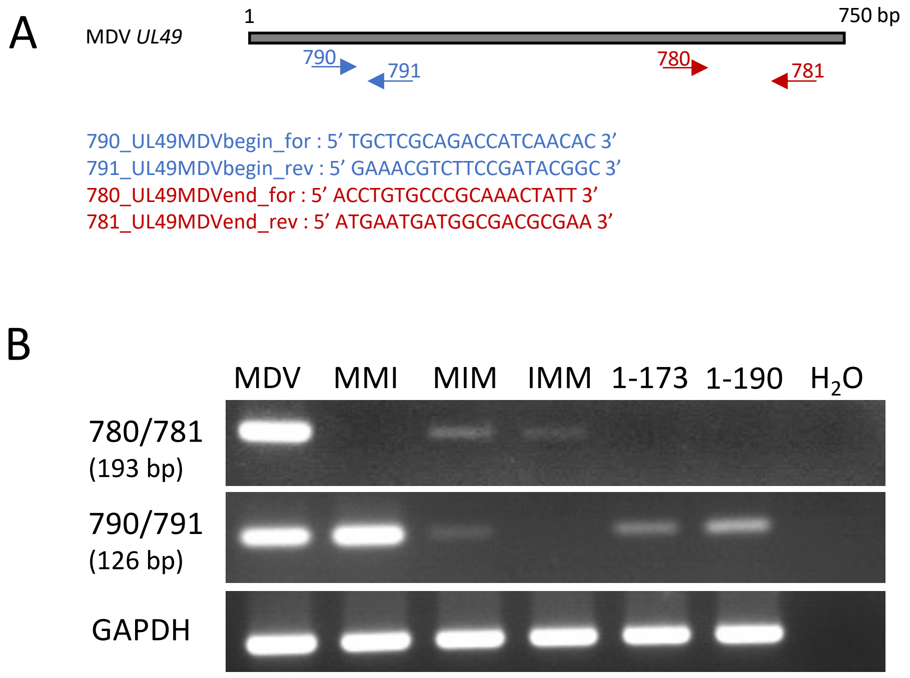

Figure S3. VP22 mRNA expression in CESCs transfected with rMDV and mutant BACs.

Due to the difficulty to assess the expression of the mutant VP22 by immunoblot when MDV mutants do not replicate efficiently, we verified *UL49* mRNA expression by reverse transcriptase PCR. CESCs were transfected with the parental rMDV BAC or mutant BACs: r22MMI, r22MIM, r22IMM, r22MDV$^{1\text{-}173}$ or r22MDV$^{1\text{-}190}$. At 72h post-transfection, total RNA were extracted using the RNeasy minikit (Qiagen) and reverse transcriptase PCR were performed as previously described (Trapp-Fragnet *et al.*, 2014). In order to detect *UL49* chimeras, two primer pairs specific to the 5′ and 3′ regions of the gene were used (A). GAPDH primers were depicted previously (Trapp-Fragnet *et al.*, 2014). All VP22 mutant genes are expressed in cells but at different levels. The MMI *UL49* mRNA expression is similar to the native *UL49* (rMDV), whereas the mRNA expression of MIM *UL49*, IMM *UL49*, UL49$^{1\text{-}173}$ and UL49$^{1\text{-}190}$ is significantly lower, which reflects the null or reduced level of replication of the corresponding mutant viruses in cells.
